# Cardiovascular and Orthostatic Responses to a Festive Meal Associated With Alcohol in Young Men

**DOI:** 10.3389/fphys.2019.01183

**Published:** 2019-09-30

**Authors:** Delphine Sarafian, Nathalie Charrière, Claire Maufrais, Jean-Pierre Montani

**Affiliations:** Laboratory of Integrative Cardiovascular and Metabolic Physiology, Faculty of Science and Medicine, University of Fribourg, Fribourg, Switzerland

**Keywords:** cardiovascular, festive meal, alcohol, postprandial, orthostasis

## Abstract

**Aim:** Sharing a festive meal associated with alcohol is quite common. While the cardiovascular changes occurring after meal ingestion of different nutrient composition has been well-established, the effects of ingesting a festive versus a standard meal accompanied with alcohol are less clear. Here, we compared the postprandial hemodynamics, cutaneous and psychomotor performance responses after ingestion of a classical Swiss festive meal [cheese fondue (CF)] versus a light ready-meal [Nasi Goreng (NG)], both accompanied with white wine.

**Methods:** In a randomized cross over design, we examined in 12 healthy young men, the continuous cardiovascular, cutaneous, and reaction time responses to ingestion of cheese fondue versus a standard meal at rest (sitting position) and hemodynamic changes in response to orthostatic challenge (active standing) in pre- and postprandial phases.

**Results:** Breath alcohol concentration after wine ingestion was similar after both meal types. Compared to the standard meal, consumption of CF induced higher increases in heart rate (HR), cardiac output (CO), double product (DP) and cardiac power output (CPO), greater vasodilation, and rises in skin blood flow and skin temperature. Greater increases in HR, DP, and mean blood pressure (MBP) were observed during orthostatic challenges with CF compared to NG. A two-choice reaction time task revealed similar reaction times with both meals, suggesting no influence of meal composition on psychomotor performance.

**Conclusion:** In sitting position, CF ingestion induced a more important cardiovascular load compared to NG. Although the dose of alcohol and the festive meal used here did not lead to orthostatic hypotension, eating CF induced a greater cardiometabolic load suggesting that hemodynamic reserves have been encroached during active standing. This may impede the cardiovascular capacity during physical exercise or stress situations, particularly in elderly subjects who are at greater risk for postprandial hypotension and cardiovascular diseases.

## Introduction

The simple act of eating induces a multitude of physiological reactions in our body such as cardiovascular and metabolic changes that are both centrally and peripherally driven. The cardiovascular responses to feeding and the role of the sympathetic nervous system activation after meal ingestion of different size and composition have been extensively reviewed (Kearney et al., 1995; van Baak, 2008). One of the hemodynamic responses that occurs postprandially is the redistribution of blood flow, with a pooling of blood in the abdominal vasculature (Lipsitz et al., 1993) and a rise in mesenteric blood flow especially after a high-fat meal (Sidery et al., 1991). A drop in systemic peripheral resistance accompanies this phenomenon but is usually compensated in healthy young subjects by rises in heart rate and cardiac output (Fagan et al., 1986; Hauser et al., 2016). Festive meals rich in fat and calories are known to induce gastric discomfort (Parker et al., 2017). In addition, compared to a low-fat meal, the consumption of a single high-fat meal could exacerbate cardiovascular reactivity during behavioral stress tasks (Jakulj et al., 2007).

Because festive meals are usually taken with alcoholic beverages, the postprandial effects of alcohol need to be taken into consideration. Ethanol induces autonomic and cardiovascular alterations such as diminished vagal tone to the heart (Koskinen et al., 1994; Spaak et al., 2010), and decreased baroreflex function (Abdel-Rahman et al., 1987). Ingestion of a mixture of wine and sugary juice reduced total peripheral resistance over time and increased skin hand blood flow (Sarafian et al., 2018). Previous results from our lab showed that compared to water or alcohol alone, drinking alcopops (alcohol with sugars) induced vasodilation and hypotension in sitting position (Maufrais et al., 2017). Moreover, ingestion of alcopops reduced the hemodynamic reserve during orthostatic challenge (Maufrais et al., 2017). Alcohol consumption can be detrimental during postural changes as it elicits hypotension related to an impairment in the vasoconstrictor response to orthostatic stress (Narkiewicz et al., 2000; Carter et al., 2011).

Consumption of cheese fondue during winter or at festive celebrations is widespread in Switzerland but also popular throughout the world (Spence, 2018). Usually accompanied with white wine, this traditional and energy-dense convivial meal has rarely been the object of investigation, except one article comparing the effects of cheese fondue associated with different types of drinks on gastric emptying and dyspeptic symptoms (Heinrich et al., 2010). Indeed, these authors highlighted a slowing down of gastric emptying when cheese fondue was ingested with white wine in comparison with black tea or water (Heinrich et al., 2010). However, to the best of our knowledge, no studies have evaluated the cardiovascular, cutaneous, and psychomotor effects of consuming cheese fondue associated with alcohol.

Here, we report the effects of alcohol consumption with a typical Swiss festive meal, i.e., cheese fondue (CF), versus a standardized light ready-meal Nasi Goreng (NG) on breath alcohol levels, cardiovascular and cutaneous responses at rest (sitting position) as well as during orthostatic tests (active standing), and psychomotor performance *via* reaction time tasks. We hypothesized that compared to a standard meal, the intake of cheese fondue, a high-fat and energy dense meal, in combination with alcohol may: (1) attenuate the rise in alcoholaemia due to expected delay in gastric emptying; (2) put an additional challenge to the cardiovascular system, which may compromise orthostatic tolerance during active standing; and (3) impair alertness and motor control/execution (increased reaction time).

## Materials and Methods

### Subjects

Twelve healthy young men were recruited from our local university student population to participate in the present study. The mean (± SEM) age of the participants was 23.3 ± 0.9 years, weight 77 ± 4.8 kg, and body mass index 23.9 ± 1.2 kg/m^2^. All subjects were Caucasian, of non-Asian ancestry, weight stable, normotensive, non-smokers, and drink alcohol only occasionally in a social context (thus excluding abstainers and chronic drinkers). None were taking medications or reported lactose-intolerance. All subjects were instructed to abstain from caffeinated drinks and alcoholic beverages 24 h before the study and to maintain their habitual diet (no dieting or overeating) and physical activity (without structured high intensity exercise). Before enrollment in the study, the participants completed a questionnaire regarding their medical history and lifestyle, and familiarized themselves with the experimental procedures and equipment. After voiding the bladder, anthropometric and body composition measurements were performed by measuring height and body weight using a mechanical column scale with integrated stadiometer (Seca model 709, Hamburg, Germany), and using a multi-frequency bioelectrical impedance analyzer (Inbody 720, Biospace Co., Ltd., Seoul, Korea). All participants gave written informed consent in accordance with the Declaration of Helsinki. The study was approved by the Swiss ethics committee on research involving humans (Canton de Vaud, CER-VD, protocol n°105/15).

### Experimental Design and Protocol

**Figure 1:**
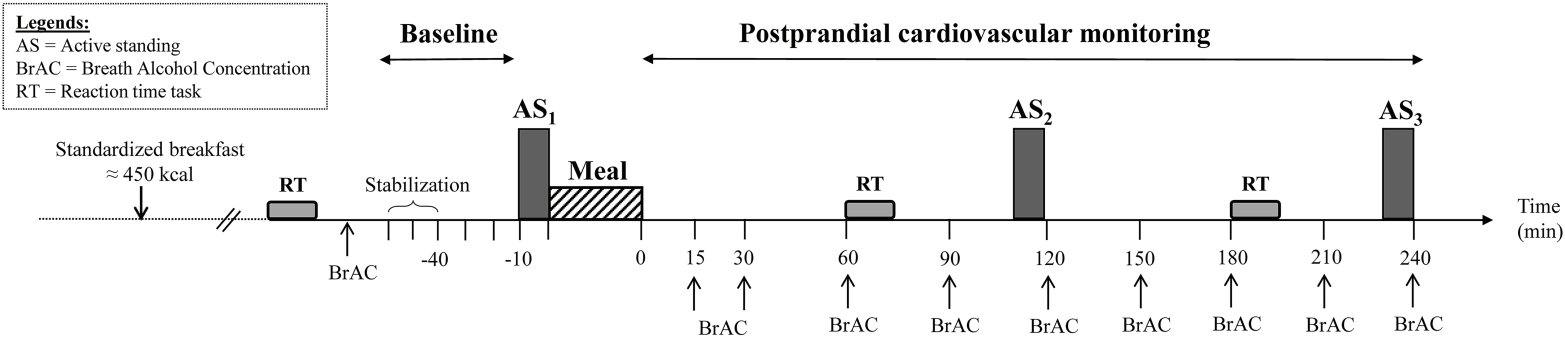
Experimental design. During the test, the participants were in the sitting position except during the three phases of active standing (AS_1_, AS_2_, and AS_3_) performed before and at 110 and 230 min after meal ingestion. Breath alcohol concentration, reaction time tasks, and thermographic pictures were measured at different time points during the test.

The study design is presented in Figure 1. Every subject attended two sessions (separated by at least 2 full days) in a quiet and temperature-controlled (21–23°C) room according to a randomized, crossover design. On the day of testing, participants were instructed to eat a 450 kcal standardized breakfast between 7:00 h and 8:00 h, provided by us and consisted of two slices of white toast bread, 15 g of butter, 30 g of jam, and 25 cl of orange juice. On arrival at the laboratory at 11:30 h, the subjects emptied their bladders and sat in a comfortable armchair while equipment for cardiovascular and cutaneous monitoring was connected. After a period of reaching cardiovascular stability (usually within 20 min), a baseline recording was conducted for 30 min. Then, the subjects performed an orthostatic test consisting of 10 min of active standing (AS) from the sitting position, with the hand held by a shoulder scarf and maintained at heart level, and then returned to sitting position. Subsequently, each subject received in random order, one of the following meals: (1) a Swiss cheese fondue (250 g + 1 dL of white wine) and (2) a commercially Nasi Goreng ready-meal. Both meals were accompanied by 3 dL of white wine and ingested over 30 min. Hemodynamic and cutaneous monitoring continued for another 240 min, with two additional orthostatic tests occurring in the postprandial period, at 110 min and 230 min after meal ingestion During each session, a 10-min break between 120 and 130 min post-meal was allowed for the participants to empty their bladder if necessary.

### Test Meals

Nutrient composition of the cheese fondue (CF) and the standard Nasi Goreng (NG) meal are presented in Table 1. The festive high-fat meal consisted of 250 g of fondue “moitié-moitié” (fondue “de la région”, Migros Switzerland), made of 50% of Gruyère and 50% of Vacherin Fribourgeois Swiss cheeses (4100 kJ/981 kcal), 175 g of white bread (1898 kJ/454 kcal). With respect to the traditional recipe, 1 dL of white wine (85 kcal) was heated until simmering after which the cheese was slowly added to the cast iron fondue pot and melted at a mean temperature of 63°C (range: 57–69°C), and stirred with a wooden spoon until all ingredients were amalgamated. The standard meal was a commercially Nasi Goreng ready-meal (370 g, 1988 kJ/474 kcal, Anna’s best, Migros, Switzerland), a mix of rice, chicken, and vegetables, which was heated for 4 min in the microwave (mean temperature: 51°C, range 49–53°C) before serving. Both meals were served with 3 dL of white wine (12.1% v/v of ethanol; 1066 kJ/255 kcal, Fendant de Sion, Valais, Denner, Switzerland) at a temperature of 14°C, which was consumed evenly over the 30 min of the meal.

**Table 1:** Nutrient composition of the Nasi Goreng and Fondue test meals.

	Nasi Goreng	Fondue
Meal: Energy content (kcal/kJ)	474/1988	1520/6352*
Carbohydrates (g)	59	87.3
Fat (g)	14	71.7
Proteins (g)	20	90.6
Drink: Energy content (kcal/kJ)	255/1066	255/1066
Total Energy content (kcal/kJ)	729/3054	1775/7418

* *The energy content of fondue meal includes 1 dL of white wine (85 kcal)*.

### Cardiovascular Recordings

As previously described in detail (Brown et al., 2005; Maufrais et al., 2018), non-invasive cardiovascular and electrocardiographic (cardiac intervals) recordings were performed using the Task Force Monitor (CNSystems, Medizintechnik, Graz, Austria). In brief, continuous beat-to-beat values of cardiac intervals and their reciprocal heart rate (HR) were recorded by ECG. Continuous systolic blood pressure (SBP) and diastolic blood pressure (DBP) were recorded alternatively from either the index or middle finger of the right hand and was automatically calibrated to oscillometric brachial blood pressure measurements (every 5 min) on the contralateral arm that was maintained on a height-adjustable table at heart level. Cardiac stroke volume (SV) was derived on a beat-to-beat basis from the impedance cardiogram. Cardiac interval variability was assessed by power spectral analysis. High frequency (HF: 0.15–0.40 Hz) power components of RR intervals (HF_RRI) were assessed in order to have an index of parasympathetic activity (Pagani et al., 1997). First given in absolute values (ms^2^), these data were processed after natural logarithmic transformation (Ln_HF RRI). Cardiac baroreflex sensitivity (BRS) was determined from spontaneous fluctuations in blood pressure and cardiac interval using the sequence method (Bertinieri et al., 1985).

### Cutaneous Recordings: Skin Blood Flow and Skin Temperature

Skin blood flow was assessed non-invasively and continuously by laser Doppler flowmetry (Perimed, Periflux System PF5001, Järfälla, Sweden), with the probe set on the dorsum of the left hand between the thumb and the index finger, as described previously (Girona et al., 2014). Skin temperature was measured in triplicate using the infrared camera FLIR E6 (FLIR Systems) at different locations of the body (dorsum of the left hand, extremity of the middle finger and nose). Thermic pictures were made at different time points throughout the test (every 10–15 min during baseline and at 15, 30, 45, 60, 90, 105, 135, 150, 165, 180, 210, and 225 min) after meal ingestion. The pictures were analyzed using the FLIR Tools software (version 6.1, FLIR Systems) at specific regions of interest as described previously (Maufrais et al., 2017).

### Breath Alcohol Concentration

Due to a very high correlation between ethanol concentration in blood and in breath (Jones and Andersson, 2003), we measured breath alcohol concentration (BrAC) by an ethylometer (Model 6820, Dräger SA, Germany) to derive blood alcohol concentration (‰ units) at baseline and at 15, 30, 60, 90, 120, 150, 180, 210, and 240 min after meal ingestion. Mean BrAC between 15 and 240 min post-ingestion (BrAC_[15–240]min_) was calculated as the area under the concentration-time curve (AUC) divided by 225 min, with AUC computed by the trapezoid method. To get a reliable BrAC measurement, the subjects were instructed to blow into the alcotest continuously during at least 5 s. At the end of each session, BrAC was measured to ensure that the subjects left the lab well below the Swiss legal limit of 0.5 ‰.

### Choice Reaction Time Task

To see whether meal type associated with alcohol can impact human behavior, in particular, the cognitive processes of perception and motor response execution, the participants performed a two-choice reaction time task before baseline, and after 1 and 3 h post-meal. Two electrodes were positioned on the muscle interosseous dorsalis I of the right hand and connected to an amplifier and a computer to record the electromyographic and mechanical responses. The subjects were sitting in front of the manipulandum composed of two different colored (red and green) LED and press buttons underneath, with their index placed midway between the two press buttons (starting position). Then, a red or a green LED lightning (visual stimulus) was randomly presented to the subjects. According to the color of the stimulus (red or green light), the subject had to execute a movement toward the corresponding colored button and press on it as quickly as possible. Before recording, each participant performed a practice block of five trials to be familiarized with the procedure. For each testing period, 36 visual stimuli were presented to the participants. The outcomes, i.e., the electromyographic and the mechanical latencies (response speed), were recorded in milliseconds using Biopac Student Lab PRO software (BIOPAC Systems, Inc., Goleta, California).

### Data Processing

Cardiovascular (cardiac intervals, systolic and diastolic blood pressure) and cutaneous parameters were first processed per minute and then averaged in 10-min intervals during baseline and throughout the 4-h postprandial period. For all cardiovascular and cutaneous variables, the changes from baseline were calculated as the absolutes values averaged over 10 min intervals to which were subtracted the 30-min of baseline measurement and presented as delta (Δ). During orthostasis, data were displayed on a 1-min basis over the 10-min of active standing. We also calculated the delta change (relative to the 4 min before standing) of cardiovascular parameters at each orthostasis for NG and CF. Heart rate was calculated by the appropriate RR interval. Cardiac output (CO) was computed as the product of stroke volume and heart rate. Mean arterial blood pressure (MBP) was calculated from DBP and SBP as follow: MBP = DBP + 1/3 (SBP − DBP). Total peripheral resistance (TPR) was calculated as MBP divided by CO. Double product (DP) was calculated as the product of HR and SBP, providing valuable information about the oxygen consumption of the myocardium (van Vliet and Montani, 1999). Stroke work was calculated as the product of systolic blood pressure and stroke volume. Cardiac power output (CPO), a measure of the cardiac pumping ability, was calculated as MBP × CO/451 (Fincke et al., 2004).

### Statistical Analysis

Data are expressed as mean ± SEM. Statistical analysis was performed by two-way ANOVA for repeated measures with time and treatments (Fondue and Nasi Goreng) as within-subject factors, using statistical program (Statistix version 8.0, Analytical Software, St. Paul, MN, USA). When significant differences were found, the effects of each treatment over time were analyzed by comparing values at each time point over the postprandial period with the average baseline value recorded during the 30-min before meal ingestion. Dunnett’s multiple comparison *post hoc* testing was used to test the changes over time relative to baseline. Difference in BrAC between the two test meals was tested by using Student paired *t* test. The level of significance for statistics was set at *p* < 0.05 (two-tailed). Repeated measures Anova followed by Dunnett’s *post hoc* analysis was performed to see the changes in cardiovascular absolute values relative to the 4 min preceding each orthostasis. To detect the effect of meal condition on orthostatic responses, we compared the changes in MBP, HR, CO, TPR, and DP (10 min average during orthostasis minus 4 min before each orthostasis) with a paired *t* test (ΔNG versus ΔCF).

## Results

### Breath Alcohol Levels

**Figure 2:**
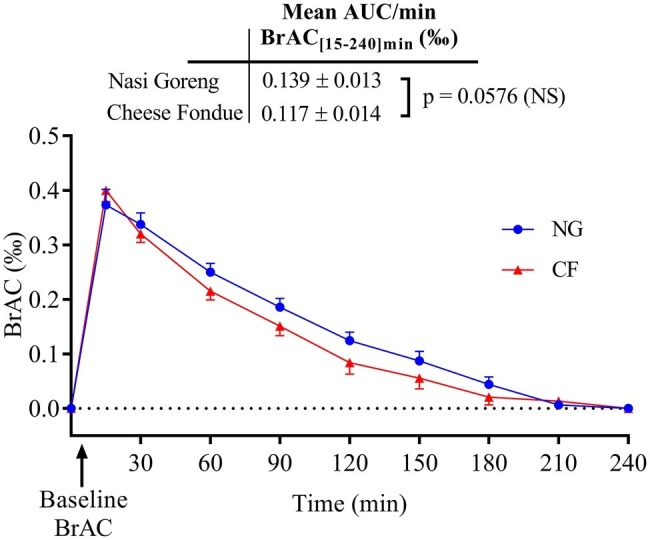
Time course of changes in breath alcohol concentration (BrAC) after wine consumption with Nasi Goreng and cheese fondue meal. The calculated mean BrAC_[15–240]min_ (‰) (± SEM) refers to the area under the concentration-time curve of BrAC between 15 and 240 min divided by 225 min after drink ingestion. Abbreviations: AUC, area under the curve; NG, Nasi Goreng; CF, cheese fondue; NS, not significant.

Changes in BrAC after meal and wine ingestion in 12 subjects are presented in Figure 2. Fifteen minutes after each meal, the BrAC levels increased to around 0.4‰. Then, the levels decreased at the end of the test until baseline levels (0‰), similarly with both test meals. Measured at several intervals over time, BrAC levels were not significantly different whether alcohol was drunk with CF or NG meal. The area under the concentration-time curve (BrAC_[15–240]min_) tended to be lower with CF (0.117 ± 0.014‰) compared to NG meal (0.139 ± 0.013‰), reaching almost statistical significance (*p* = 0.0576).

### Cardiovascular Responses to Meal Ingestion

Baseline hemodynamic and cutaneous characteristics (prior to meal ingestion) are summarized in Table 2.

**Table 2:** Basal cardiovascular and cutaneous parameters of 12 participants recorded on two separate test days.

Variable	Nasi Goreng	Fondue
Systolic blood pressure (mmHg)	115.5 ± 2.2	114.6 ± 1.8
Diastolic blood pressure (mmHg)	73.7 ± 2.5	74.0 ± 1.5
Mean blood pressure (mmHg)	87.6 ± 2.4	87.8 ± 1.7
Heart rate (beats.min^−1^)	65.6 ± 3.0	67.4 ± 2.6^*^
Stroke volume (mL)	78.9 ± 3.3	77.3 ± 3.4
Cardiac output (L.min^−1^)	5.11 ± 0.18	5.16 ± 0.21
Total peripheral resistance (mmHg.min.L^−1^)	17.4 ± 0.8	17.2 ± 0.6
Double product (mmHg.beats.min^−1^)	7,604 ± 410	7,747 ± 362
Baroreflex sensitivity (ms.mmHg^−1^)	17.3 ± 2.0	17.5 ± 1.7
HF_RRI (ms^2^)	570 ± 268	593 ± 267
Ln HF_RRI (Ln ms^2^)	5.76 ± 0.29	5.74 ± 0.31
Stroke work (mmHg.mL)	9,080 ± 346	8,854 ± 405
Cardiac power output (watt)	0.99 ± 0.04	1.00 ± 0.05
Skin blood flow (AU)	26.9 ± 3.0	26.2 ± 3.2
Hand temperature (°C)	36.4 ± 0.4	35.6 ± 0.9
Finger temperature (°C)	36.4 ± 0.6	35.5 ± 1.1
Nose temperature (°C)	36.5 ± 0.6	35.9 ± 0.8

*Data are mean ± SEM*.

* *p = 0.04, significant difference between Nasi Goreng and Fondue*.

*HF_RRI, high-frequency power components of RR internals; Ln, natural logarithmic transformation; AU, arbitrary units*.

**Figure 3:**
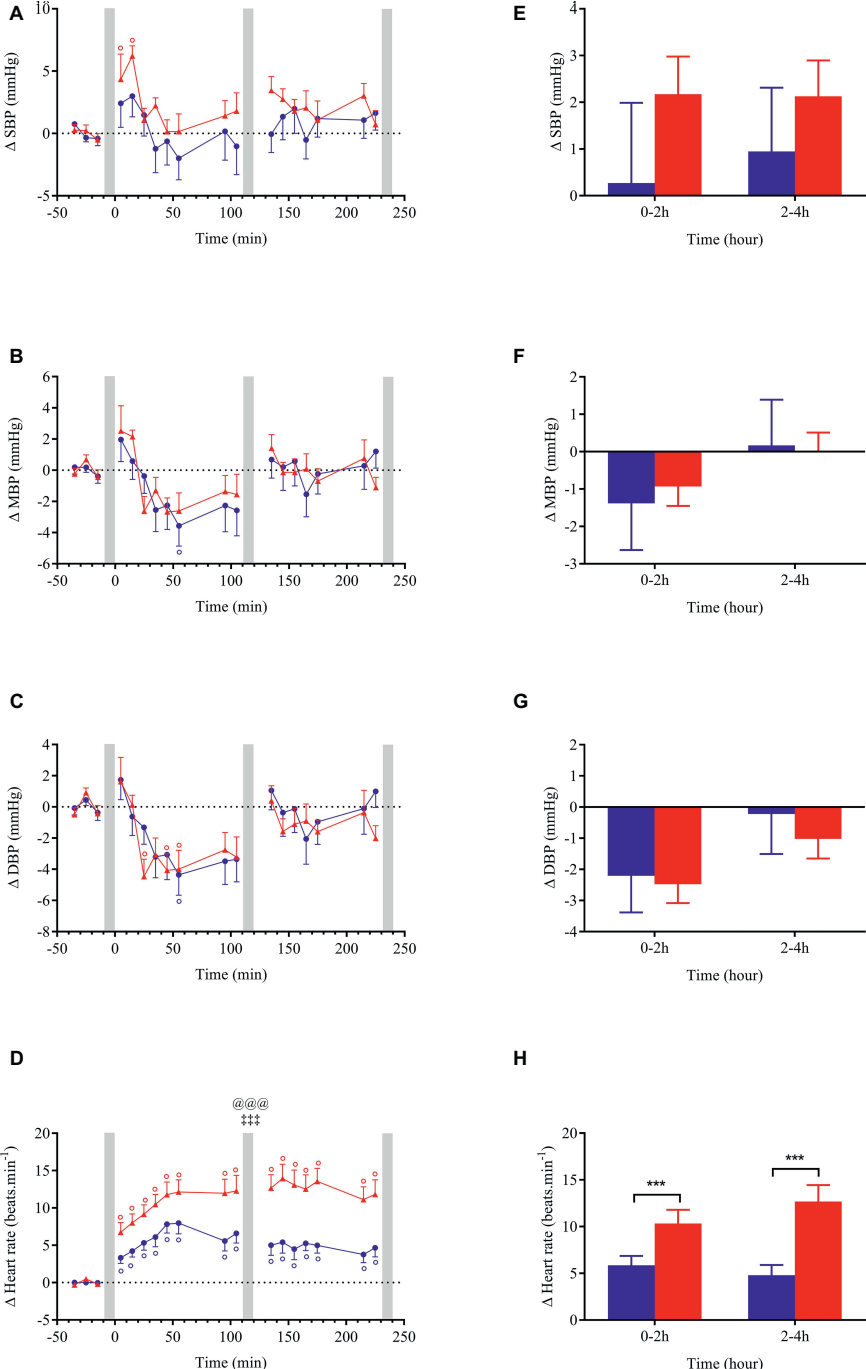
Left panels **(A–D)**: time course of changes in systolic blood pressure (SBP), mean blood pressure (MBP), diastolic blood pressure (DBP) and heart rate in response to meal ingestion and presented as delta (i.e., absolute changes relative to baseline). Right panels **(E–H)**: mean changes averaged over 0–2 h and 2–4 h after meal ingestion and presented as delta (i.e., average over 0–2 h and 2–4 h post-meal minus the average over the 30-min baseline period, respectively) for the same parameters. Test meals: NG = Nasi Goreng (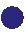, 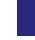), CF = cheese fondue (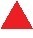, 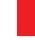). Values are mean ± SEM. Symbols for Anova repeated measures analysis: @ meal effect, ‡ time × meal interaction effects. °Significant difference over time from baseline values (Dunnett’s test). Histograms: *significant difference between meal conditions (NG vs. CF) by paired *t*-test. Level of significance was mentioned as follow: 1 symbol (*p* < 0.05), 2 symbols (*p* < 0.01), 3 symbols (*p* < 0.001). Grey vertical bars represent orthostasis challenges.

**Figure 4:**
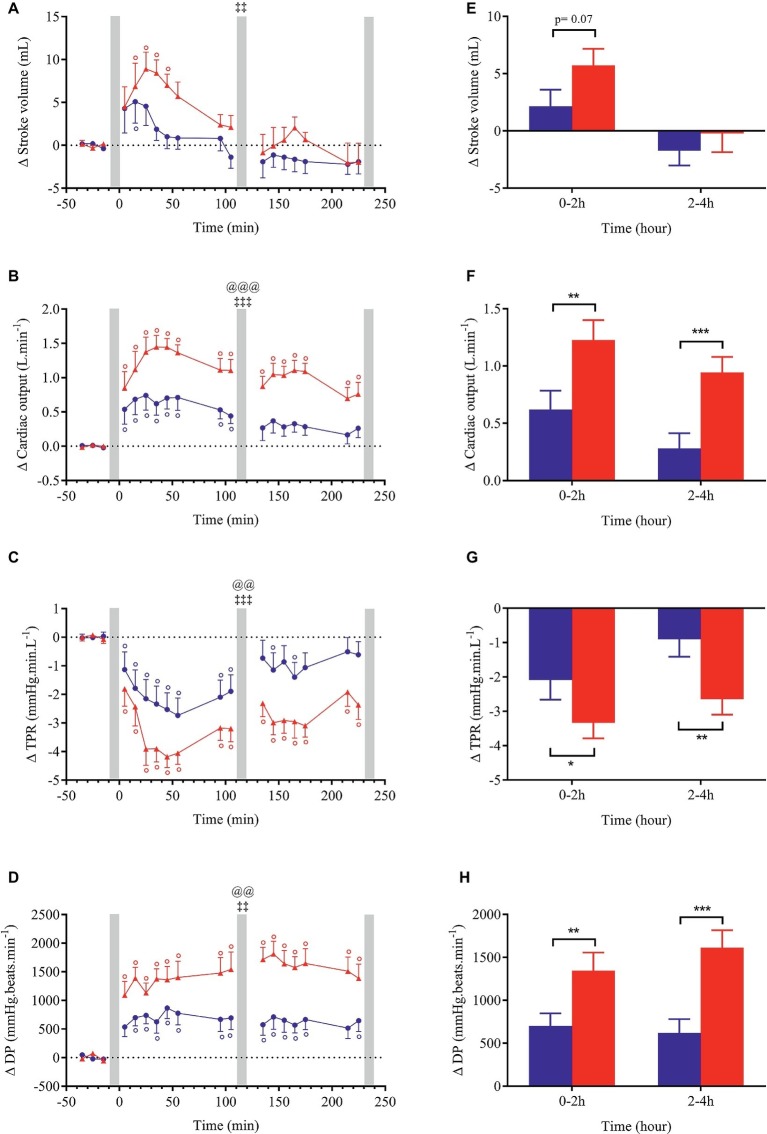
Left panels **(A–D)**: time course of changes in stroke volume, cardiac output, total peripheral resistance (TPR) and double product (DP) in response to meal ingestion and presented as delta (i.e., absolute changes relative to baseline). Right panels **(E–H)**: mean changes averaged over 0–2 h and 2–4 h after meal ingestion and presented as delta (i.e., average over 0–2 h and 2–4 h post-meal minus the average over the 30-min baseline period, respectively) for the same parameters. Test meals: NG = Nasi Goreng (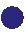, 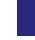), CF = cheese fondue (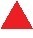, 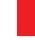). Values are mean ± SEM. For statistical symbols, see legends of Figure 3.

**Figure 5:**
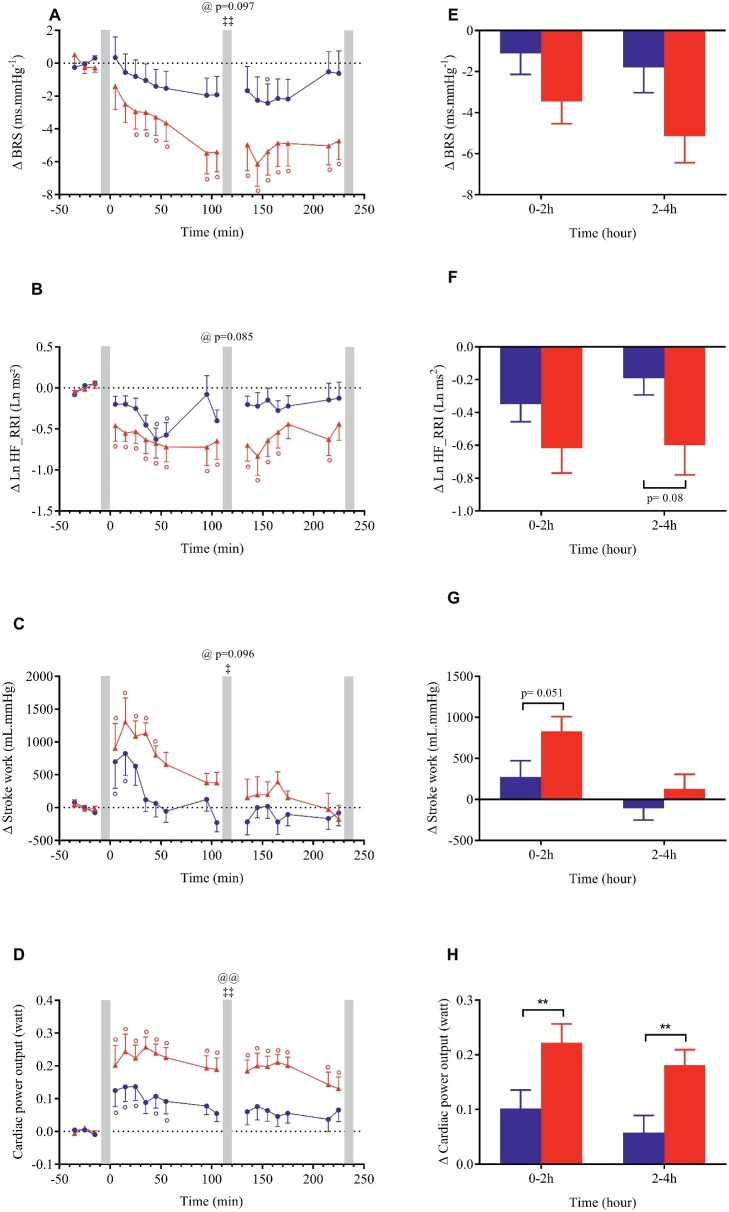
Left panels **(A–D)**: time course of changes in baroreflex sensitivity (BRS), natural logarithmic transformation of high-frequency power components of RR intervals (Ln HF_RRI), stroke work and cardiac power output in response to meal ingestion and presented as delta (i.e., absolute changes relative to baseline). Right panels **(E–H)**: mean changes averaged over 0–2 h and 2–4 h after meal ingestion and presented as delta (i.e., average over 0–2 h and 2–4 h post-meal minus the average over the 30-min baseline period, respectively) for the same parameters. Test meals: NG = Nasi Goreng (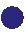, 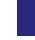), CF = cheese fondue (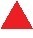, 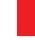). Values are mean ± SEM. For statistical symbols, see legends of Figure 3.

**Figure 6:**
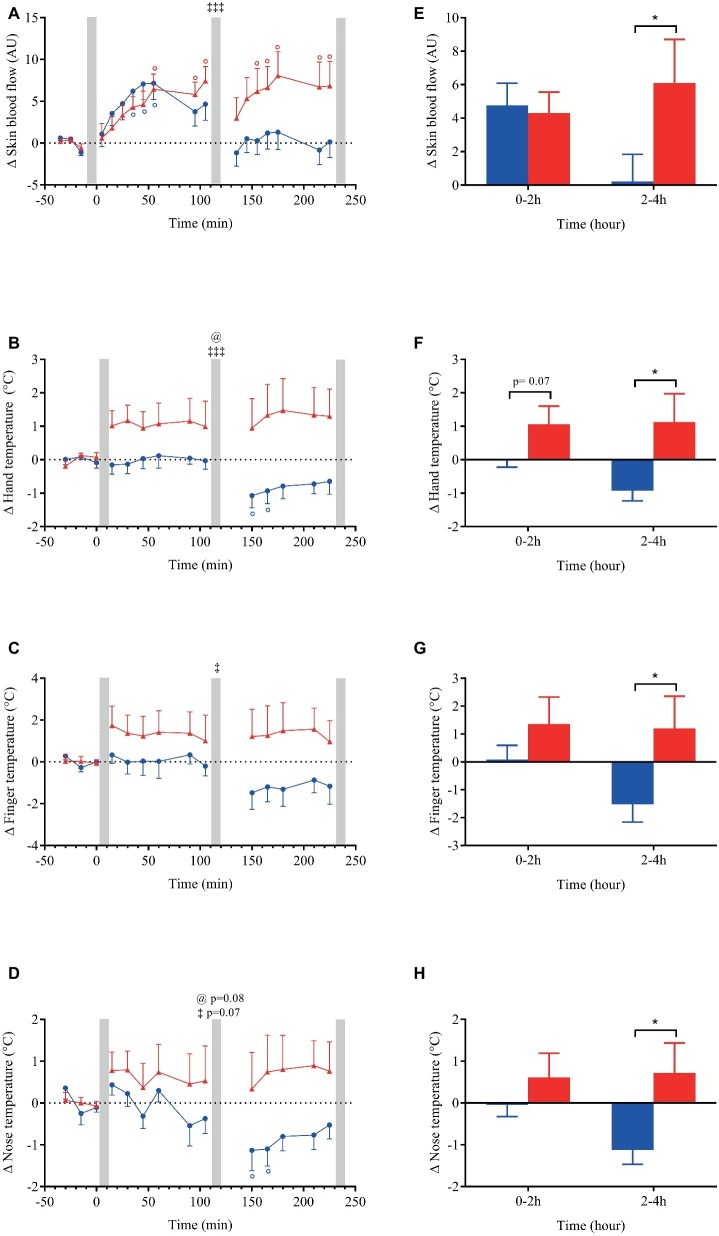
Left panels **(A–D)**: time course of changes in skin blood flow, skin hand, finger and nose temperature in response to meal ingestion and presented as delta (i.e., absolute changes relative to baseline). Right panels **(E–H)**: mean changes averaged over 0–2 h and 2–4 h after meal ingestion and presented as delta (i.e., average over 0–2 h and 2–4 h post-meal minus the average over the 30-min baseline period, respectively) for the same parameters. Test meals: NG = Nasi Goreng (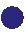, 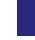), CF = cheese fondue (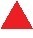, 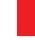). Values are mean ± SEM. For statistical symbols, see legends of Figure 3.

Changes (relative to baseline) for all cardiovascular and cutaneous variables are shown in Figures 3–6. Ingestion of the standard meal and the cheese fondue meal with wine resulted in significant time × meal interaction effects for all variables studied except for SBP, DBP, MBP, Ln HF_RRI, and nose temperature. Meal effect was observed for HR, CO, TPR, DP, CPO, and hand temperature parameters.

Changes in blood pressure after meal ingestion were minimal with no significant difference in MBP, only a very slight increase in SBP with CF in the first 30 min and a small decrease in DBP (<4.5 mmHg) in the first postprandial hour with both meals (Figures 3A–C, E), leading to a small increase in pulse pressure that tended to be greater with CF meal (*p* = 0.07, data not shown). In contrast, heart rate increased significantly above baseline values (*p* < 0.001) after both meals with a greater magnitude with CF than for NG between 0 and 2 h (ΔHR_NG_ = +5.9 vs. ΔHR_CF_ = +10.3 beats.min^−1^; Figure 3H) and between 2 and 4 h after meal ingestion (ΔHR_NG_ = +4.8 vs. ΔHR_CF_ = +12.7 beats.min^−1^). Eating CF induced an increase in stroke volume during the first hour after meal (Figures 4A,E) and a two-fold increase in CO and DP during the entire postprandial period, compared to NG (Figures 4B,D,F,H). In parallel, TPR dropped immediately after both test meals (*p* < 0.001, Figures 4C,G) and remained significantly lower with CF compared to NG at 0–2 h (ΔTPR_CF_ = −3.3 vs. ΔTPR_NG_ = −2.1 mmHg.min.L^−1^) and 2–4 h post-meal (ΔTPR_CF_ = −2.6 vs. ΔTPR_NG_ = −0.9 mmHg.min. L^−1^).

Despite a tendency for BRS and Ln HF_RRI to be further reduced with CF compared to NG (Figures 5A,B,E,F), no significant meal effect was found. Just after CF ingestion, stroke work increased above baseline and tended to be augmented compared to NG (*p* = 0.051) between 0 and 2 h post-meal. Furthermore, CPO was largely increased with CF compared to NG (meal and time × meal interaction effects *p* < 0.01; Figures 5D,H) during the entire postprandial period.

Within the first hour post-ingestion, skin blood flow was increased after both meals but returned to baseline with NG (Figure 6A). By contrast, skin blood flow remained elevated after CF during the whole test with a clear meal effect in the late postprandial phase (*p* < 0.05). Regarding the thermic responses to meal ingestion, we observed that NG showed no change or a slight decrease in skin temperature between 2 and 4 h after meal. In contrast, ingestion of CF induced an elevation in skin hand, finger, and nose temperature that was statistically different from NG in the late phase of meal test (Figures 6B,D,F,H).

### Cardiovascular Responses to Orthostatic Challenges

**Figure 7:**
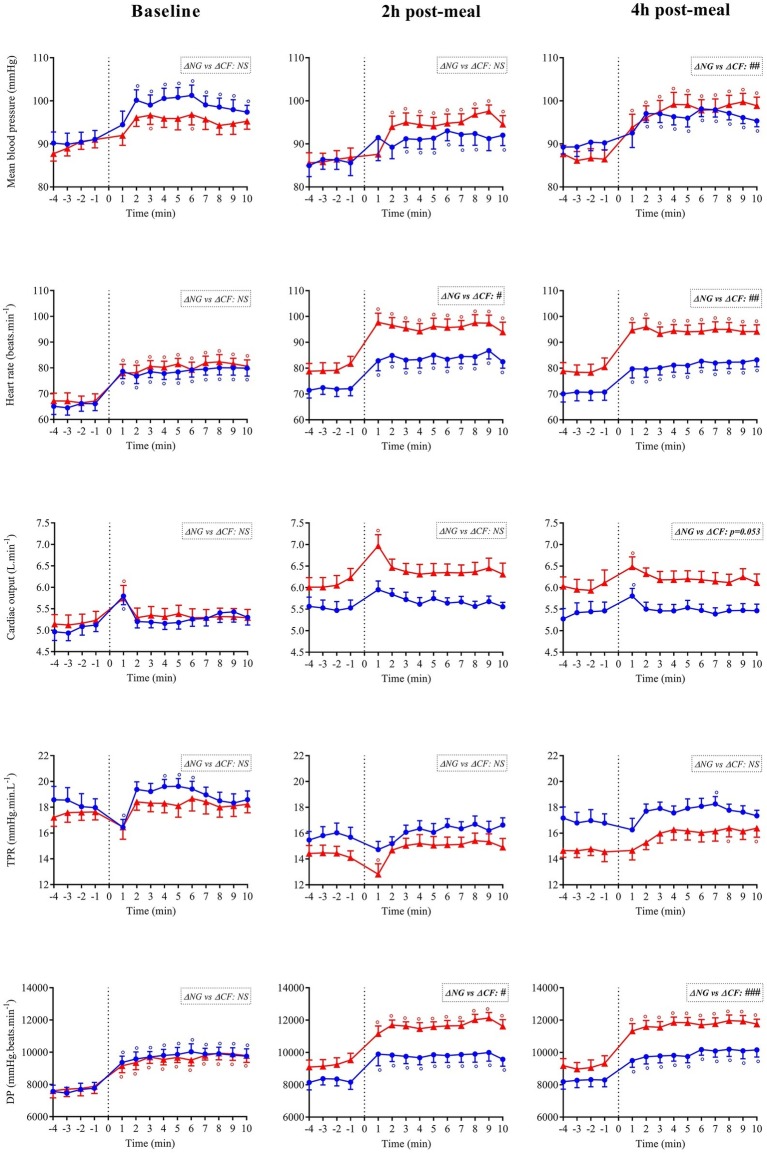
Absolute values of mean blood pressure, heart rate, cardiac output, total peripheral resistance (TPR), and double product (DP) during orthostatic challenges (active standing) performed at baseline, at ~2 and ~4 h after ingestion of Nasi Goreng (NG, 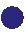) and cheese fondue (CF, 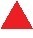). Data are presented minute per minute, during the 4 min preceding standing position and during the 10 min of orthostatic challenges. The box at the top right refers to the statistical comparison of the changes in all variables (i.e., average during the 10 min of active standing minus the 4 min just before orthostasis) between NG and CF during orthostasis. °Significant difference over time from baseline values (Dunnett’s test). #Significant difference between NG and CF during active standing after paired *t* test. The level of significance is shown as follow: #(*p* < 0.05), ##(*p* < 0.01) and ###(*p* < 0.001).

The cardiovascular responses to orthostatic challenges for MBP, HR, CO TPR, and DP are presented in Figure 7. Data were expressed in absolutes values in order to bring to light the global cardiovascular load of orthostatic tests performed before and after meal. Before ingestion of either meal, the variables followed the same trend in response to active standing with no significant difference between meal conditions for all parameters. At 2 h post-meal, the cardiovascular changes (delta relative to the 4 min preceding standing) were qualitatively similar in both meals but they were clearly amplified after eating CF compared to NG for HR (+16.4 vs. +12.1 beats.min^−1^), DP (+2400 vs. +1513 mmHg.beats.min^−1^) and CO (+0.35 vs. +0.16 L.min^−1^) and were maintained 4 h after meal ingestion. A greater blood pressure response was observed with CF at 4 h post-meal.

### Electromyographic and Mechanical Latencies in Response to a Choice Reaction Time Task

For each meal condition, we observed similar electromyographic and mechanical latencies across the three reaction time tasks performed at baseline, 1 and 3 h after meal and alcohol ingestion (Table 3).

**Table 3:** Electromyographic (EMG) and mechanical latencies (milliseconds) during a choice reaction time task performed at baseline, 1 and 3 h after meal ingestion.

	Meal	Baseline	1 h Post-meal	3 h Post-meal	Anova
EMG latency (ms)	Nasi Goreng	230 ± 9	238 ± 11	239 ± 9	NS
	Fondue	239 ± 6	230 ± 9	236 ± 8	NS
Mechanical latency (ms)	Nasi Goreng	381 ± 18	380 ± 18	371 ± 16	NS
	Fondue	383 ± 10	378 ± 10	371 ± 11	NS

*Data are mean ± SEM. Anova repeated measures analysis was performed across baseline, 1 and 3 h post-meal. NS, not significant*.

## Discussion

Because drinking alcohol with a meal is a common social situation, we investigated whether the type of meal ingested (light versus energy-dense) in combination with alcohol has a different impact on the cardiovascular, cutaneous, and psychomotor responses. Here, we show that (1) although total alcohol intake was slightly higher with CF meal, BrAC tended to be lower compared to NG; (2) ingestion of cheese fondue, a high caloric meal, led to a greater cardiac load compared to a lighter meal, as shown by higher increases in HR, CO, DP, and CPO and to greater cutaneous responses; (3) basal increases in HR and DP during orthostatic challenges were amplified after CF compared to NG; and (4) the type of meal ingested had no influence on psychomotor performance as assessed by reaction time task.

### Effect of Meal Type on Breath Alcohol Concentration

As described elsewhere, BrAC reflects blood alcohol concentration since these two parameters are well correlated (Pikaar et al., 1988; Jones and Andersson, 2003). In various culinary preparations containing alcohol, a part of the alcohol is lost by evaporation but a significant amount of alcohol can be retained (Augustin et al., 1992) depending on several factors such as heat applied, time of cooking and the size of cooking vessel. In our study, since the temperature of CF was maintained below the bowling point of pure ethanol (<78.5°C at 1 bar), a significant loss of alcohol by evaporation was not expected in the cheese fondue. However, despite a greater alcohol intake in CF meal, BrAC tended to be lower compared to NG meal. This could be related to a greater delay in gastric emptying, as observed by others after a high-fat meal (Houghton et al., 1990; Sidery et al., 1994), thereby promoting ethanol metabolism by the gastric mucosa (Frezza et al., 1990; Jones and Jonsson, 1994). This is consistent with studies showing that drinking alcohol following the consumption of a high-energy meal produced a smaller area under the curve of BrAC compared to a low-energy meal (Pikaar et al., 1988) or after alcopops, i.e., drinks mixing alcohol and sucrose (Maufrais et al., 2017).

### Cardiovascular Responses to the Ingestion of a Light Versus a Festive Meal

Our results showed that at rest, the consumption of a festive meal like cheese fondue associated with wine induced a greater postprandial cardiovascular load compared to a standard light meal, as reflected by greater increases in HR, CO, DP, stroke work, and CPO. After CF meal, cardiac vagal tone tended to be reduced in the late postprandial phase. Indeed, BRS and Ln_HF RRI were lower relative to baseline (*p* < 0.05) and close to be statistically different to NG meal (*p* = 0.08, Figure 5F). This result converged with the parasympathetic withdrawal (decreased high-frequency power of heart rate variability) seen after the intake of a high-energy and high-fat meal (Kuwahara et al., 2011).

In our study, the cardiovascular changes in response to meal ingestion seem to be dependent on meal composition and energy content (Table 2). In accordance with our results, greater increases in HR and CO were observed following the ingestion of a larger breakfast (3 MJ) compared to a smaller one (1 MJ) (Sidery and Macdonald, 1994). Moreover, the proportion of macronutrients ingested may contribute to this effect because compared to water intake, a high-energy liquid meal (6.8 MJ) rich in fat and sugars caused a paralel increase in HR and CO accompanied with a decrease in vascular resistance (Hauser et al., 2016). Protein content can also contribute to the increase in HR and CO (Avasthi et al., 1987; Waaler and Eriksen, 1992). Finally, sodium intake may be implicated in the augmented cardiac responses since acute salt loading caused a significant increase in HR and mean arterial pressure in healthy young adults (Charkoudian et al., 2005).

Our results showed a more pronounced fall in TPR with CF compared to NG, without corresponding changes in blood pressure. This is consistent with previous study showing a greater vasodilation after ingestion of a meal containing the highest caloric load (3 versus 1 MJ) (Sidery and Macdonald, 1994), indicating that energy content of meal could influence the magnitude of vascular peripheral resistance. The maintenance of MBP in our study may be explained by a greater CO increase in response to CF compared to NG meal. The blood pressure responses observed in our study are in accordance with other studies showing no alteration in blood pressure after eating a 6.3 MJ standard meal (Kelbaek et al., 1989), a 6.8 MJ high-fat meal (Hauser et al., 2016), or a 2.5 MJ high-carbohydrate meal (Sidery et al., 1991).

The greater cardiac load in our study after CF ingestion may be attributed to higher proportions of the three main macronutrients (fat, proteins, and carbohydrates), and of salt combined with the 2.4-fold greater energy content of CF compared to NG meal (7.4 versus 3.1 MJ, respectively). Knowing that protein-rich meals are more thermogenic than sugary or fatty-rich meals (Johnston et al., 2002; Tentolouris et al., 2008), the co-ingestion of high levels of proteins, fat, sugars and alcohol contained in CF may be responsible for the overactivation of the cardiovascular system to ensure metabolic processes associated with digestion and food absorption.

### Cutaneous and Thermic Responses to the Ingestion of a Light Versus a Festive Meal

In our study, skin blood flow increased similarly with both meals during the first 2 h post-meal and remained significantly elevated in the late postprandial phase with CF meal. The difference in the time course of blood flow between meals may be due in part by delayed gastric emptying after ingesting rich-fat meals (Houghton et al., 1990; Sidery et al., 1994) and to a global higher caloric load with CF. Previous studies have shown that ingesting a fatty meal (Raitakari et al., 2000) and oral glucose loading (Forst et al., 1998) caused vasodilator effects and increases in skin microvascular blood flow, respectively which seems to be associated with postprandial insulin, C-peptide, and triglyceride levels. In our study, we observed a significant difference in finger, hand and nose temperature that were higher with CF only in the late postprandial phase. A recent study has reported an increase in mean skin temperature following the ingestion of a standardized liquid meal (Martinez-Tellez et al., 2019). The warmer skin temperature observed after the festive meal is assumed to be the consequence of a greater thermic effect of food (metabolic rate and body temperature) in response to a heavy and energy-dense meal like CF.

### Orthostatic Responses After Ingestion of a Light Versus a Festive Meal

To mimic festive situations where standing up intermittently in the postprandial state is a usual practice, we tested the effects of a light versus heavy meal on the orthostatic responses. In the postprandial phase, we clearly observed accentuated cardiovascular responses to active standing at 2 and 4 h following CF ingestion compared to NG, in particular greater absolute values of HR, CO, DP, and MBP that can be viewed as a first encroach into the hemodynamic reserve. Even when looking only at the changes from the 4 min preceding orthostasis (delta), we observed a greater increase in HR and DP after CF ingestion. Vascular resistance decreased with postprandial time after CF ingestion but was relatively stable during active standing without effect of meal type. Previous results from our lab showed that maintenance of the standing position induced a rise in HR in the fasting state (Miles-Chan et al., 2013), or after alcohol ingestion (Maufrais et al., 2017). Moreover, HR was higher during repeated standing (vs. sitting) after consuming a high-fat mixed meal (Altenburg et al., 2019) or during repeated standing (vs. supine) after a carbohydrate-rich meal (Cao and Pilowsky, 2014). Our data showed the shifting up of HR, CO, and DP values 4 min before orthostatic test as well as during active standing periods, with CF showing the greatest increase. Although consumption of both meals did not induce postprandial hypotension, the hemodynamic reserve has been encroached due to cardiovascular overload induced by the CF high-caloric meal.

### Psychomotor Performance Assessed by Reaction Time Tasks

The effects of major macronutrients on cognitive functions have already been the object of investigation (Hoyland et al., 2008) with a positive impact of carbohydrates on mental performance (Dye et al., 2000). Little attention has been given to the impact of fat or alcohol taken singly or in combination on psychomotor performance. In our study, the combination of a high-fat meal and alcohol intake did not impair the reaction time in comparison with a light meal. This could be explained by relatively low alcoholaemia already observed at 1 and 3 h after meal. This does not exclude that higher BrAC levels could impair performance. A higher alcoholaemia level (~0.5‰) induced longer reaction time and slowed information processing (Tzambazis and Stough, 2000). Slower reaction time was reported after high-carbohydrate or high-fat compared to medium-fat/medium-carbohydrate lunches (Lloyd et al., 1994), while others found no effect of meal type (high-carbohydrate vs. protein) on performance (Finnigan et al., 1998) and no evidence of fat content or meal size influencing performance of vigilance tasks (Smith et al., 1994). Here, the absence of potentiation of meal and alcohol on the reaction time might be explained by a relatively low total dose of alcohol and the meal-induced lowering of blood alcohol levels.

## Conclusion And Perspectives

The present study suggested that compared to a light meal (NG), the consumption of an energy-dense festive meal (CF) induced a greater cardiovascular load in sitting position. Furthermore, the responses to active standing after a festive meal showed a reduction of cardiac reserve lasting at least 4 h post-ingestion. Since BrAC levels were similar in both meals, the cardiovascular and cutaneous responses seem more related to the nutritional composition and caloric content of CF or to the interaction of the festive meal and alcohol. Even in healthy young men, eating a heavy meal in combination with alcohol is likely to increase cardiac load at rest and encroach the hemodynamic reserve during orthostatic challenges in the postprandial phase.

This first study was limited to men but should be extended to women, healthy elderly subjects and to people suffering from cardiovascular diseases. Indeed, knowing that heavy meals (Trahair et al., 2014) and alcohol consumption (Wolk et al., 2001) can be linked to postprandial hypotension with syncopal events particularly in elderly, individuals with cardiovascular dysfunction such as heart failure or hypertension could be at greater risk after a high-fat meal associated with alcohol. Moreover, as cardiac reserve is diminished after a festive meal during standing position, it may impede the cardiovascular capacity during a physical exercise or during stress situations of higher energy demand. Vascular and cutaneous responses after a high-fat meal raise also the question of the tolerance of festive meals during heat stress, which may be detrimental for individuals at risk for cardiovascular diseases.

## Data Availability

The datasets generated for this study are available on request to the corresponding author.

## Ethics Statement

The studies involving human participants were reviewed and approved by Swiss ethics committee on research involving humans (Canton de Vaud, CER-VD). The patients/participants provided their written informed consent to participate in this study.

## Author Contributions

DS performed data analysis, statistics, drafted the manuscript and contributed to interpretation of data, and critical revision of the work for important intellectual content. NC recruited subjects, performed data acquisition and analysis. CM assisted to data acquisition, contributed to interpretation of data, and critical revision of the work for important intellectual content. J-PM conceived and designed the research, contributed to interpretation of data, and critical revision of the work for important intellectual content.

### Conflict of Interest Statement

The authors declare that the research was conducted in the absence of any commercial or financial relationships that could be construed as a potential conflict of interest.
